# Drug-Drug Interactions between COVID-19 and Tuberculosis Medications: A Comprehensive Review of CYP450 and Transporter-Mediated Effects

**DOI:** 10.3390/ph17081035

**Published:** 2024-08-06

**Authors:** M. Rasheduzzaman Jony, Sangzin Ahn

**Affiliations:** 1 Department of Pharmacology and Pharmacogenomics Research Center, Inje University College of Medicine, Busan 47392, Republic of Korea; mdrasheduzzamanjony226@gmail.com; 2 Center for Personalized Precision Medicine of Tuberculosis, Inje University College of Medicine, Busan 47392, Republic of Korea

**Keywords:** COVID-19, anti-tuberculosis, drug-drug interactions, enzyme, transporter

## Abstract

Most medications undergo metabolism and elimination via CYP450 enzymes, while uptake and efflux transporters play vital roles in drug elimination from various organs. Interactions often occur when multiple drugs share CYP450-transporter-mediated metabolic pathways, necessitating a unique clinical care strategy to address the diverse types of CYP450 and transporter-mediated drug-drug interactions (DDI). The primary focus of this review is to record relevant mechanisms regarding DDI between COVID-19 and tuberculosis (TB) treatments, specifically through the influence of CYP450 enzymes and transporters on drug absorption, distribution, metabolism, elimination, and pharmacokinetics. This understanding empowers clinicians to prevent subtherapeutic and supratherapeutic drug levels of COVID medications when co-administered with TB drugs, thereby mitigating potential challenges and ensuring optimal treatment outcomes. A comprehensive analysis is presented, encompassing various illustrative instances of TB drugs that may impact COVID-19 clinical behavior, and vice versa. This review aims to provide valuable insights to healthcare providers, facilitating informed decision-making and enhancing patient safety while managing co-infections. Ultimately, this study contributes to the body of knowledge necessary to optimize therapeutic approaches and improve patient outcomes in the face of the growing challenges posed by infectious diseases.

## 1. Introduction

The coronavirus disease 2019 (COVID-19) pandemic, caused by the severe acute respiratory syndrome coronavirus 2 (SARS-CoV-2), has affected over 113 million individuals and resulted in more than 2.5 million deaths worldwide, as reported by the World Health Organization (WHO). Both tuberculosis (TB) and COVID-19 are infectious diseases that predominantly target the lungs. Co-infection with TB and COVID-19 has been associated with a significant increase in mortality rates, with some studies reporting an alarming 11.86-fold increase compared to patients solely affected by COVID-19 [1]. Other studies have found that the mortality rate in TB and COVID-19 co-infected patients is 12.3%, which is 5.3 times higher than the mortality rate observed in patients solely infected with COVID-19 [2].

To address the urgent need for effective treatment, several drugs have been proposed as potential interventions to mitigate the progression of clinical conditions. Remdesivir (RDV), an RNA-dependent RNA polymerase (RdRp) inhibitor, stands out as the first effective antiviral that has been discovered and approved for the treatment of COVID-19 [3,4]. However, due to its parenteral administration, RDV is primarily used to treat hospitalized patients. To expand treatment options for outpatients and reduce the risk of severe consequences, such as hospitalization and death, the U.S. Food and Drug Administration (FDA) has granted emergency use authorization to two medications: molnupiravir (Lagevrio, Merk, Rahway, NJ, USA) and a combination of nirmatrelvir and ritonavir (paxlovid, Pfizer, Hong Kong). These drugs are now approved for treating mild-to-moderate SARS-CoV-2 infections in individuals at a high risk of developing severe COVID-19, including hospitalization or death [4].

The interaction between COVID-19 treatments and anti-TB drugs is a significant concern, as drug-drug interactions (DDIs) can pose challenges to the widespread use of these medications. In many healthcare settings, FDA-approved COVID-19 therapies are prescribed primarily by practitioners who may lack extensive knowledge of managing complex DDIs, particularly those associated with anti-TB drugs. This knowledge gap can inadvertently lead to treatment denial or serious adverse events due to unintended DDIs. To ensure safe management, it is crucial for prescribers to not only be aware of drugs that are contraindicated for use with FDA-approved COVID-19 treatments but also to know which medications can be safely co-administered. This knowledge helps prevent unnecessary denial of treatment or inappropriate withholding of long-term comedications, promoting better patient care.

The purpose of this review is to provide a comprehensive summary of the effects of anti-TB drugs on drug disposition, including their impact on metabolizing enzymes and transporters. Additionally, we discuss various factors that influence the likelihood of clinically significant DDIs between FDA-approved anti-COVID-19 and anti-TB drugs. By understanding these factors, healthcare professionals can make informed decisions to minimize the risk of adverse events and optimize treatment outcomes. Furthermore, this review addresses the management of selected DDIs of interest, providing practical guidance to clinicians in handling potential interactions between anti-TB drugs and FDA-approved COVID-19 therapies. By synthesizing this information, our review aims to support healthcare providers in making informed and safe treatment decisions, ensuring effective management of both COVID-19 and TB, while minimizing the risks associated with drug interactions.

## 2. COVID-19 and Tuberculosis Co-Infection

The COVID-19 pandemic has posed unprecedented challenges to healthcare systems worldwide. As of March 2024, the SARS-CoV-2 virus has infected over 113 million people and claimed more than 2.5 million lives. The pandemic has also exposed the vulnerabilities of healthcare systems in managing co-infections, particularly in patients with pre-existing conditions such as TB.

Tuberculosis, an infectious disease caused by Mycobacterium tuberculosis, remains a significant global health concern. In 2019, an estimated 10 million people fell ill with TB, and 1.4 million died from the disease [5]. The emergence of COVID-19 has further complicated the management of TB, as both diseases primarily affect the lungs and can lead to severe respiratory complications.

The development of COVID-19 treatments has is a priority for researchers and pharmaceutical companies worldwide. Following the reporting of the complete viral genome, the 3-dimensional structure of the SARS-CoV-2 major protease (Mpro) was rapidly determined [6]. SARS-CoV-2 Mpro closely resembles the Mpro of SARS-CoV-1, and insights from previous research on other viruses, such as picornaviruses and the hepatitis C virus, facilitated the identification of potential protease inhibitors. This has accelerated the search for effective treatments against COVID-19.

Several drugs have been repurposed or developed to treat COVID-19, including remdesivir (RDV), an RNA-dependent RNA polymerase (RdRp) inhibitor, and a combination of nirmatrelvir and ritonavir (paxlovid), which targets the viral protease. Additionally, molnupiravir, a prodrug of the active compound beta-D-N4-hydroxycytidine (NHC), has shown promise as an orally administered antiviral agent [7,8].

However, the use of these COVID-19 treatments in patients with TB raises concerns regarding potential DDIs. Anti-TB drugs, such as rifampin, isoniazid, and pyrazinamide, are known to interact with various metabolic enzymes and transporters, which can significantly impact the pharmacokinetics and pharmacodynamics of co-administered medications [9]. These interactions can lead to reduced efficacy or increased toxicity of COVID-19 treatments, compromising patient outcomes.

To optimize treatment strategies and minimize the risk of adverse events, it is essential to understand the role of metabolic enzymes and transporters in drug disposition and the factors that influence the likelihood of clinically significant DDIs. This knowledge will enable healthcare professionals to make informed decisions when managing patients with TB and COVID-19 co-infections, ultimately improving patient care and outcomes.

## 3. Role of Metabolic Enzymes and Transporters in Drug Disposition

Drug-drug interactions (DDIs) are significantly influenced by metabolic enzymes, particularly cytochrome P450 (CYP) enzymes [10,11]. Hepatic CYP enzymes are impacted by the prior administration of other medicines, which accounts for many of the significant pharmacokinetic interactions between pharmaceuticals [12]. Drug responsiveness, interactions, and adverse effects can all be significantly impacted by CYP450 metabolism. When a strong CYP450 enzyme inhibitor or inducer is administered concurrently with medications that are metabolized by one or more CYP450 enzymes, patients should be closely monitored for the emergence of adverse pharmacological effects or therapeutic failures. Drugs with a high extraction ratio are typically less impacted by CYP450 interactions than those with a low extraction ratio. Both in vivo and in vitro methods for determining drug-drug interactions are practical [12].

Medications can impact the CYP450 system in numerous ways. For example, metoprolol is metabolized by the CYP2D6 enzyme, while warfarin [Coumadin] is processed by the CYP1A2, CYP2D6, and CYP3A4 enzymes [13]. Inhibitors impede the metabolic activity of one or more CYP450 enzymes. Depending on the dose and the inhibitor’s ability to bind to the enzyme, inhibitors have varied effects on the metabolism of a medication. Sertraline, for example, is thought to be a modest inhibitor of CYP2D6 at a dose of 50 mg, but at a dose of 200 mg, it becomes a potent inhibitor. Most inhibitory effects start to take effect immediately. A drug can also both inhibit and be metabolized by the same enzyme at the same time (as erythromycin does), or it can induce its own metabolism (like terbinafine does) [14]. It is possible to purposely combine medications in order to gain from CYP450 inhibition. Ritonavir, a protease inhibitor and potent CYP3A4 inhibitor, is used with lopinavir to raise serum levels in patients with human immunodeficiency virus. To develop strategies for reducing the risks brought on by these interactions and to better understand the role of metabolic enzymes in drug-drug interactions, more research is required.

On the other hand, membrane transporters, particularly those expressed in tissue barriers and the liver, the organ responsible for the majority of drug metabolism, have a significant impact on the pharmacokinetics, pharmacodynamics, toxicity, and side effects of pharmaceutical compounds. Hepatocytes’ canalicular ABC (ATP Binding Cassette) transporters, including ABCC2/MRP2, ABCG2/BCRP, ABCB1/MDR1/P-gp, and ABCB11/BSEP (bile salt export pump), mediate the extrusion of endo- and xenobiotics into the bile. P-gp, MRP2, and ABCG2 are multi-specific transporters that mediate the efflux of hydrophobic or partially detoxified amphiphilic compounds. MRP2 is the primary transporter of bilirubin conjugates. The SLC-type transporter MATE1 (SLC47A1; multidrug and toxin extrusion protein 1) in the hepatocyte canalicular membrane is primarily responsible for transporting drugs, but some zwitterionic and anionic compounds are also transported [15,16], aiding in their excretion into the bile. The inhibition of these drug exporters may exacerbate liver damage due to compound accumulation.

Many drugs have the potential to block ABCB11 (bile salt export pump, BSEP), the pump responsible for bile salt extrusion into the canaliculi, leading to cholestasis or drug-induced liver injury (DILI) [17]. By efflux of harmful substances, such as medications, into the venous circulation, basolateral/sinusoidal ABC transporters (ABCC3/MRP3 and ABCC4/MRP4) protect hepatocytes from any potential overaccumulation [18].

Important SLC-type (“uptake”) drug transporters in sinusoidal circulation include SLC22A1/OCT1, SLC10A1/NTCP, SLCO1B1/OATP1B1, and SLCO1B3/OATP1B3. OCT1, in collaboration with apical MATE1, selectively facilitates the entry of cationic medicines into hepatocytes. NTCP and BSEP are important players in the enterohepatic circulation of bile acids because they both carry out sodium-dependent bile acid absorption in the basolateral membrane of hepatocytes. NTCP is a known risk factor for hepatic drug-drug interactions because it also promotes the absorption of numerous medicines by hepatocytes, including statins [19]. OATP1B1 and OATP1B3 are the two primary organic anion uptake transporters in the liver. With comparatively different selectivities and specificities, these transporters are responsible for hepatocellular absorption and removal of a wide range of medications and hazardous chemicals from the bloodstream. When these transporters are blocked, many therapeutically utilized medicines become more hazardous overall and result in greater blood retention [20]. There is a dearth of knowledge about remdesivir (RDV), favipiravir (FAV), and ivermectin (IVE)’s transporter-related pharmacology and toxicological properties compared to lopinavir (LOP) and ritonavir (RIT) [21].

## 4. FDA-Approved COVID-19 Treatments and Their Potential DDI with Anti-TB Drugs

### 4.1. Remdesivir (RDV)

#### 4.1.1. A Brief on RDV

Remdesivir (RDV) is one of the few medications approved by the U.S. Food and Drug Administration (FDA) for the treatment of severe cases of COVID-19. The drug undergoes metabolism through both CYP (cytochrome P450) enzymes and non-CYP enzymes. Earlier research has highlighted RDV as a substrate for CYP2C8, CYP2D6, and CYP3A4 enzymes. Moreover, Aleissa et al. (2020) reported that RDV inhibits the activity of several enzymes, including CYP1A2, CYP2C9, CYP2C19, CYP2D6, and, crucially, CYP3A4. CYP3A4 is a vital enzyme responsible for metabolizing approximately 70% of the medications used in clinical settings. As a result, it is crucial to consider the potential for drug interactions when co-administering RDV with other medications that also affect CYP3A4 expression. The simultaneous use of such medications could lead to reduced elimination of RDV, resulting in unpredictable dose toxicity and potentially adverse effects. Healthcare professionals should exercise caution and closely monitor patients receiving RDV alongside other medications known to affect CYP3A4 activity. Understanding and managing these drug interactions are essential to ensure the safe and effective use of RDV in treating severe COVID-19 cases.

#### 4.1.2. Potential DDI with Anti-TB Drugs

The potential of drug-drug interactions (DDIs) involving RDV as a precipitant has not been thoroughly investigated. Based on available information, RDV exhibits weak inhibitory activity against CYP3A4, organic anion transporter protein 1B1 (OATP1B1), OATP1B3, and MATE1 [22]. However, its likelihood of being the perpetrator of clinically significant DDIs is limited by its specific route of administration, transient exposure, and rapid clearance. 

As an object of DDIs, RDV metabolism involves CYP2C8, CYP2D6, and CYP3A, with CYP3A identified as the primary enzyme responsible for hepatic metabolism, accounting for 10% of total hepatic clearance [23].

The intracellular activation pathway of RDV, mediated by esterases, is unlikely to be inhibited by commonly used drugs. RDV has been identified as a substrate for OATP1B1 and P-glycoprotein in vitro, but the impact of these transporters on RDV disposition is considered minimal due to its parenteral route of administration [22].

**Figure 1:**
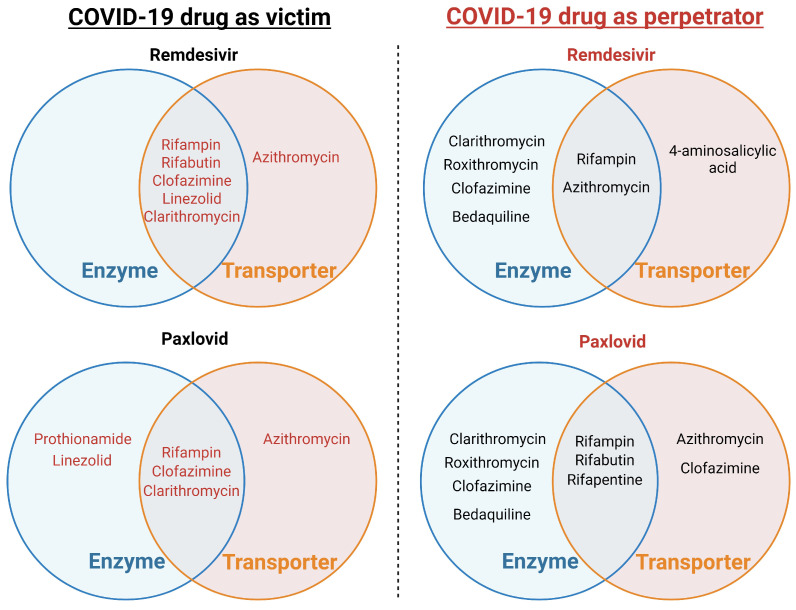
Potential CYP450 and transporter-mediated drug-drug interactions between FDA-approved COVID-19 drugs and anti-tuberculosis drugs.

Clinical DDI studies on RDV are yet to be conducted. However, a mathematical prediction of its DDI liability was performed using available in vitro data and phase I data from healthy volunteers [22]. Predictions indicate that the known levels of CYP3A induction by rifampin, a strong inducer, and a four-fold induction of esterase activity are anticipated to result in a 30% reduction in RDV exposure. Similarly, complete CYP3A inhibition is expected to increase RDV exposure by only 4%, in contrast with a 20-fold increase in midazolam exposure, a probe CYP3A substrate [22]. Given its intravenous route of administration and moderate-to-high extraction ratio, the effect of inducers or inhibitors on RDV pharmacokinetics is expected to be substantially attenuated compared to scenarios involving drugs with low hepatic extraction ratios [24]. These findings support the permissibility of weak or moderate CYP3A inducers and strong CYP3A inhibitors in the RDV phase III clinical program, with planned evaluations of the DDI between rifampin and RDV. Potential enzyme-transporter-mediated drug-drug interactions of remdesivir with anti-TB drugs as a victim and perpetrator are summarized in Figure 1, Table 1 and Table 2.

### 4.2. Paxlovid (Nirmatrelvir and Ritonavir Combination)

#### 4.2.1. A Brief on Paxlovid

Nirmatrelvir/ritonavir is a COVID-19 drug that has been approved for clinical use. As an inhibitor of the major protease, nirmatrelvir’s properties offer hope for the development of effective treatments against the virus [25]. Its administration can be carried out in a controlled hospital environment or as a one-time infusion in various outpatient settings, ensuring compliance with prior therapies. Patient adherence to their treatment plans is of utmost importance to achieve optimum effectiveness and minimize toxicities or other adverse effects.

Paxlovid, a medication comprising nirmatrelvir and ritonavir, is available in tablet form. Ritonavir is provided in 100 mg pills, while nirmatrelvir comes in 150 mg tablets. The current prescription for paxlovid entails a dose of 300 mg nirmatrelvir (two 150-mg tablets) and 100 mg ritonavir (one 100-mg tablet), to be taken orally twice daily for five days. This treatment regimen is recommended for patients 12 years of age and older, weighing at least 40 kg. For nirmatrelvir to have the desired therapeutic impact, it is essential to co-administer it with ritonavir. The combination of these two components in paxlovid is crucial to enhance the drug’s effectiveness in treating COVID-19 cases. Ensuring concurrent administration of nirmatrelvir and ritonavir is vital to achieve the desired treatment outcomes and effectively combat viral infection. Patients should strictly follow their prescribed dosing schedule to optimize therapeutic benefits and minimize the risk of treatment failure or adverse effects [25].

#### 4.2.2. Potential DDI with Anti-TB Drugs

Despite ritonavir being used for many years to treat HIV, limited information is available about its potential for drug interactions or the significance of specific interactions when combined with nirmatrelvir during a brief, five-day course of therapy for COVID-19. Nirmatrelvir has been identified as a substrate for human MDR-1 (P-gp) and CYP3A4 enzymes based on in vitro research. However, it is not a substrate for human BCRP, MATE1, MATE2K, NTCP, OAT1, OAT2, OAT3, OCT1, OCT2, PEPT1, OATPs 1B1, 1B3, or 4C1. Clinically relevant quantities of nirmatrelvir do not cause inhibition of CYP1A2, CYP2B6, CYP2C8, CYP2C9, CYP2C19, or CYP2D6. However, it has been found to reversibly inhibit CYP3A4 in a time-dependent manner, as well as P-gp.

Preliminary results from studies involving the administration of itraconazole and carbamazepine in combination with nirmatrelvir/ritonavir after five doses have been reported [23]. These studies aimed to assess the potential for drug interactions when these medications were used together during the five-day treatment regimen [25]. Given the limited available information on the interactions and significance of combining nirmatrelvir and ritonavir, further research and monitoring of patients receiving this combination therapy are necessary to better understand its implications and ensure patient safety and treatment efficacy. Potential enzyme-transporter-mediated drug-drug interactions of paxlovid with anti-TB drugs as a victim and a perpetrator are summarized in Figure 1, Table 3 and Table 4.

### 4.3. Molnupiravir

#### 4.3.1. A Brief on Molnupiravir

Molnupiravir, also referred to as EIDD-2801 or MK-4482, is an orally bioavailable prodrug with an isopropyl ester structure. Upon ingestion, it undergoes hydrolysis facilitated by host kinases in the bloodstream, converting it into beta-D-N4-hydroxycytidine (NHC), a cytidine nucleoside analog. Inside host cells, NHC is further modified through phosphorylation, ultimately forming NHC-TP, which serves as a competitive alternative for viral RdRp (ns12). As NHC-TP is integrated as NHC-monophosphate (NHC-MP) into the growing viral RNA chain, it triggers a mechanism of action known as viral error catastrophe or viral lethal mutagenesis. This mechanism involves the accumulation of errors within the viral genome, effectively hindering viral replication.

The recommended dosage of molnupiravir is 200 mg per capsule. For adults over the age of 18, the advised treatment regimen is to take 800 mg (four 200-mg capsules) orally once every 12 h for a total of five days [25]. However, it is important to note that the use of molnupiravir in children is currently not recommended. In patients diagnosed with COVID-19, therapy with molnupiravir should ideally begin no later than five days after the onset of symptoms. Molnupiravir can be administered with or without food, offering flexibility in its administration.

As a COVID-19 therapeutic drug, molnupiravir can be provided in a controlled hospital environment or administered as a one-time infusion in various outpatient settings. Ensuring compliance with prior therapies is essential to maximize treatment effectiveness and minimize the potential for toxicity or other adverse effects. To achieve the desired treatment outcomes, patients must adhere to their prescribed treatment plans precisely. Following the recommended dosage and schedule is crucial to optimize the effectiveness of molnupiravir in combating viral infection and promoting better recovery.

#### 4.3.2. Potential DDI with Anti-TB Drugs

Molnupiravir has not been associated with any significant drug interactions in humans. This is because molnupiravir is a prodrug, meaning it is converted to its active form by human esterases. As a result, the potential for drug interactions is considered to be low. In vitro tests have shown that both molnupiravir and its active form (NHC) are not substrates of CYP enzymes, human P-gp, or BCRP transporters. Additionally, they do not suppress the activity of CYP1A2, CYP2B6, CYP2C8, CYP2C9, CYP2D6, or CYP3A4, as well as transporters like OATP1B1, OATP1B3, MATE1, MATE2K, OAT1, OAT3, OCT1, OCT2, and BCRP. Furthermore, molnupiravir and NHC do not induce the activity of CYP1A2, CYP2B6, or CYP3A4 [25]. These in vitro findings suggest that the risk of drug interactions with molnupiravir is minimal, providing an additional advantage for its use in COVID-19 therapy. However, it is essential to continue monitoring any potential drug interactions during the clinical use of molnupiravir to ensure patient safety and treatment efficacy. As with any medication, healthcare professionals should exercise caution and carefully evaluate a patient’s medication regimen when considering the addition of molnupiravir to their treatment plan.

## 5. Clinical Implications and Knowledge Gaps

The current evaluation emphasizes the potential risks associated with using certain medications to treat COVID-19 and TB. Both COVID-19 and TB drugs are metabolized by CYP enzymes and transported by ABC and SLC transporters [24]. The role of these transporters in drug disposition can significantly impact drug-drug interactions, particularly for antiviral drugs. Although there has been substantial research on drug-drug interactions and the role of CYP enzymes in commonly used medications for COVID-19 and TB, there are still several drugs with unknown interactions with limited clinical data [26].

Changes in transporters and drug-metabolizing enzymes (DMEs) can alter the pharmacokinetic properties of the drugs, affecting both their efficacy and toxicity [27]. Therefore, understanding the role of enzymes and transporters is crucial for determining the overall effectiveness and safety of medications. 

It is essential to note that drug interactions should not be disregarded because they are often avoidable and can be beneficial. Combining medications like nirmatrelvir/ritonavir can lead to better treatment outcomes [28]. However, certain drug combinations, such as remdesivir and baricitinib, have shown promise in improving clinical status but have also resulted in significant side effects [29]. Additionally, the use of multiple medications simultaneously increases the likelihood of drug interactions. This is particularly important to consider, as some medications have been associated with the potential for developing torsades de pointes (TdP) [30], a type of heart rhythm disorder. Furthermore, there have been instances of moderate-to-severe QTc prolongation observed during various COVID-19 and TB treatment regimens.

From the above discussion, a potential knowledge gap arises concerning the comprehensive understanding of DDIs involving medications used to treat COVID-19 and TB infections, including limited clinical data. Although there has been substantial research on the role of CYP450 enzymes and ABC and SLC transporters in commonly used medications, there are still several drugs with unknown interactions. The specific drug interactions involving these medications, especially in combination with each other, remain incompletely characterized.

Further research is needed to elucidate how various COVID-19 and TB drugs interact with CYP enzymes and transporters, both in vitro and in vivo. This knowledge is essential for guiding treatment decisions and ensuring patient safety, as drug interactions can significantly impact the efficacy and safety of therapeutic regimens. Additionally, more research is required to assess the potential risks and benefits of combination therapies, such as nirmatrelvir/ritonavir, and the influence of these combinations on treatment outcomes.

Understanding the potential for QTc prolongation and other adverse effects associated with multiple medication regimens used in COVID-19 and TB treatment is also crucial for tailoring treatment plans to individual patients. Furthermore, given the complexity of drug interactions and the involvement of various enzymes and transporters, pharmacogenomic studies may shed light on individual variability in drug metabolism and response. Integrating pharmacogenomic data into clinical practice could enable personalized medical approaches and optimize treatment strategies for different patient populations.

Addressing these knowledge gaps will facilitate the development of evidence-based guidelines for selecting and combining medications effectively and safely for the treatment of COVID-19 and TB infections. It will also help healthcare providers make informed decisions, minimize the risk of adverse events, and maximize the treatment efficacy for better patient outcomes.

While choosing optimal treatment strategies for individual patients, healthcare professionals must carefully consider the potential for drug-drug interactions. Understanding the role of enzymes and transporters in drug metabolism can aid in the selection of appropriate medications to achieve better treatment outcomes while minimizing the risks associated with drug interactions. Monitoring patients for adverse effects and accordingly adjusting their treatment plans can further enhance their safety and treatment efficacy.

## 6. Implication of PBPK Modeling Approach to Facilitate the Clinical Decision-Making

Physiologically based pharmacokinetic (PBPK) modeling is a sophisticated and robust tool used to predict DDIs between anti-COVID-19 and anti-TB therapies. This modeling approach offers several significant implications for clinical practice and drug development.

PBPK models integrate physiological, biochemical, and drug-specific data to simulate the absorption, distribution, metabolism, and excretion (ADME) of drugs. By incorporating parameters such as enzyme kinetics and transporter interactions, PBPK models can predict the potential of DDIs at various dosage levels [31,32]. This is particularly crucial for co-administered anti-COVID-19 and anti-TB drugs, in which interactions can lead to altered therapeutic efficacy or increased toxicity. PBPK modeling can aid in optimizing dosing regimens to mitigate the risk of adverse DDIs [33]. For instance, by simulating different dosing scenarios, healthcare providers can identify dosing strategies that minimize interactions and maintain drug efficacy. This is especially important in managing co-infections like COVID-19 and tuberculosis, where patients may require simultaneous treatment with multiple drugs. The ability of PBPK models to account for individual patient characteristics, such as age, weight, organ function, and genetic polymorphisms, supports personalized medicine [34]. This individualized approach ensures that patients receive tailored treatment regimens that are both safe and effective, reducing the risk of DDIs and improving overall treatment outcomes. PBPK models are increasingly being used in regulatory submissions to predict potential DDIs during drug development. Regulatory agencies, including the FDA and EMA, recognize the value of PBPK modeling in providing evidence for safe and effective drug use [35,36]. By predicting DDIs early in the development process, pharmaceutical companies can make informed decisions regarding drug candidate selection and clinical trial design. PBPK modeling provides insights into the mechanistic pathways of drug interactions [37]. For example, it can elucidate how anti-TB drugs like rifampicin, which is a potent inducer of cytochrome P450 enzymes, interact with anti-COVID-19 drugs metabolized by the same enzymes. Understanding these pathways will help in developing strategies to avoid or manage DDIs. Several PBPK models have been developed to assess the potential DDIs of paxlovid [38,39,40]. An initial PBPK model using Simcyp™ indicated that paxlovid’s inhibition of CYP3A-mediated midazolam clearance and P-gp-mediated dabigatran etexilate transport was primarily attributed to ritonavir, as only minor differences in DDI magnitude were observed between paxlovid and ritonavir [39]. Further PBPK models suggested necessary dosing adjustments for the elexacaftor-tezacaftor-ivacaftor combination used in cystic fibrosis and for rivaroxaban, an anticoagulant, due to CYP3A inhibition by paxlovid. These studies highlight the value of PBPK modeling in early DDI risk assessment [38,40]. Additionally, PBPK modeling has been employed to explore the victim DDI potential of remdesivir, an intravenous antiviral initially developed for ebola treatment [22,41]. 

**Figure 2:**
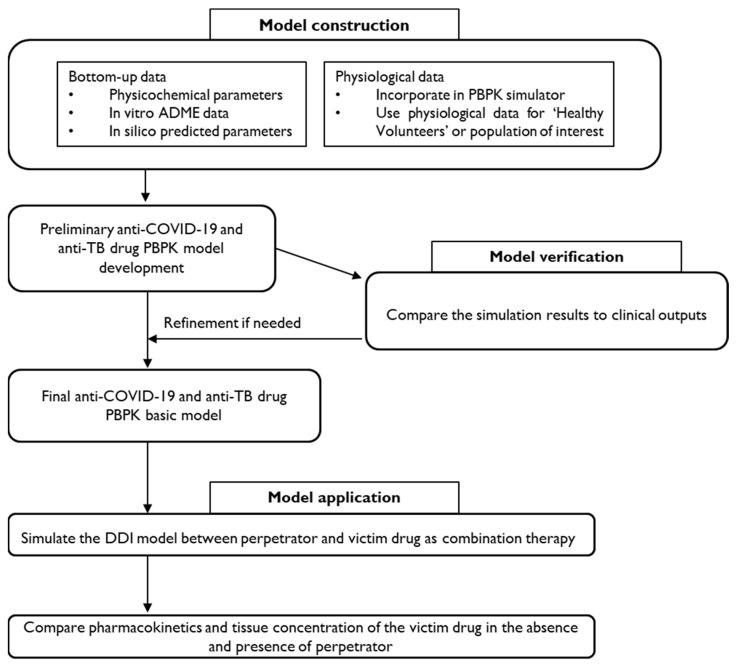
A proposed framework for PBPK-DDI model development for anti-COVID-19 and anti-TB drugs.

A basic flowchart for the development of the PBPK-DDI model is illustrated in Figure 2.

Table 5 provides the range of essential drug-related parameters required for the PBPK-DDI models. In addition to physiological and drug-specific properties, the end user can specify various DDI trial parameters, including the dose of each drug, frequency of administration, pre- or post-dosing intervals, and route of administration [42,43].

Clinicians can use PBPK model outputs to make informed decisions regarding drug selection and dosing in patients with co-infections. For instance, PBPK models can simulate the impact of adding an anti-TB drug to an existing anti-COVID-19 regimen, predicting changes in drug concentrations and potential clinical outcomes. This supports evidence-based clinical decision-making and enhances patient safety.

## 7. Recommendations for Frontline Healthcare Professionals

### 7.1. Stay Informed

Keep up to date with the latest research, guidelines, and clinical trials related to drug interactions between COVID-19 and TB medications. Regularly review reputable sources and publications to enhance knowledge of potential interactions, dosing adjustments, and safety considerations.

### 7.2. Foster Interprofessional Collaboration

Encourage collaboration among physicians, nurses, and pharmacists to optimize patient care. Regularly communicate and consult with the healthcare team to identify and manage drug interactions, ensure appropriate medication regimens, and improve patient outcomes.

### 7.3. Create Comprehensive and Personalized Treatment Plans

When managing patients with COVID-19 and TB co-infection, perform a thorough review of their medication history, including concurrent treatments. Consider patient-specific factors, such as genetic variations, comorbidities, and potential drug interactions, to create tailored treatment plans that minimize risks and maximize effectiveness.

### 7.4. Monitor and Report Adverse Events

Closely monitor patients for potential adverse effects, particularly those related to drug interactions, such as QTc prolongation. Regularly assess patients’ clinical status, laboratory results, and medication tolerance. Promptly report any suspected adverse drug reactions or unexpected side effects to relevant authorities or reporting systems.

### 7.5. Educate Patients

Provide clear and concise information to patients about their prescribed medications, including possible drug interactions, dosing instructions, and potential side effects. Encourage patients to report unusual symptoms or changes in their health during treatment.

### 7.6. Engage in Shared Decision-Making

Involve patients in treatment decisions whenever possible. Communicate the risks and benefits of specific medications, including potential interactions, to help patients make informed choices regarding their healthcare.

### 7.7. Pursue Continuous Learning

Engage in continuing education programs and training related to pharmacology, drug interactions, and the management of COVID-19 and TB infections. Stay informed about emerging research and best practices to support better decision-making in patient care.

## 8. Conclusions

The ongoing COVID-19 pandemic has posed unprecedented challenges to healthcare systems worldwide, particularly in the management of patients with co-existing conditions, such as tuberculosis. As demonstrated by our analysis of CYP450 and transporter-mediated interactions (see Table 1, Table 2, Table 3 and Table 4), the potential for drug-drug interactions between COVID-19 and TB medications is a significant concern. For example, the interaction between remdesivir and rifampin through CYP3A4 inhibition (Table 1) illustrates how these interactions can significantly impact the efficacy and safety of treatment regimens. 

PBPK modeling is crucial for predicting DDI in drug development [44]. While current efforts focus on transporters and P450-mediated interactions, there is a growing emphasis on non-P450 DDIs, enhanced by in vitro studies and clinical trials [45,46]. As PBPK models advance, data quality has become increasingly important. Efforts to refine transporter-and protein therapeutic-based models alongside traditional small-molecule model, are ongoing [47]. Developing robust bottom-up models before clinical data are available remains a priority [48]. PBPK modeling’s role in ensuring drug safety and efficacy is growing, as reflected in regulatory filings and publications.

This review article has provided a comprehensive overview of the role of metabolic enzymes and transporters in drug disposition, the potential drug-drug interactions between FDA-approved COVID-19 treatments and anti-TB drugs, and the clinical implications of these interactions.

Understanding the complex interplay between metabolic enzymes, such as cytochrome CYP enzymes, and transporters, including ABC and SLC transporters, is crucial for healthcare professionals to make informed decisions when selecting and combining medications for patients with COVID-19 and TB co-infection. While substantial research has been conducted on drug-drug interactions involving commonly used medications, there remain knowledge gaps, particularly concerning newer COVID-19 treatments and their interactions with anti-TB drugs. Further research is needed to fully characterize these interactions, both in vitro and in vivo, to guide treatment decisions and ensure patient safety.

This comprehensive review contributes significant scientific value by synthesizing the current knowledge on drug-drug interactions between COVID-19 and TB medications, with a specific focus on metabolic enzymes and transporters. By identifying critical knowledge gaps, particularly regarding newer COVID-19 treatments, and providing evidence-based recommendations, this work informs both clinical practice and future research directions. As the global healthcare community navigates the ongoing challenges of the COVID-19 pandemic, it is crucial for frontline professionals to leverage this knowledge to manage complex drug interactions. Through interprofessional collaboration, personalized treatment plans, and evidence-based strategies, healthcare providers can optimize treatment outcomes and minimize the risks for patients with COVID-19 and TB co-infection. Ultimately, this review’s insights and practical guidelines will contribute to improving patient safety and public health in the face of this global health crisis.

## Figures and Tables

**Table 1:** Potential enzyme-transporter-mediated drug-drug interactions of remdesivir (victim) with anti-TB drugs (perpetrator).

TB-Drugs (Perpetrator)	COVID Drug (Victim)	Possible Pathway	Possible Mechanism
Rifampin	Remdesivir	Transporter/enzyme interplay	Inhibition of CYP3A4, P-gp and OATP transporters or induction of metabolic enzymes and P-gp transporters
Rifabutin		Transporter/enzyme interplay	Inhibition of P-gp efflux pump and induction of CYP3A4
Clofazimine		Transporter/enzyme interplay	Inhibition of CYP3A4 enzyme and P-gp efflux pump
Linezolid		Transporter/enzyme interplay	Inhibition of CYP3A4 enzyme and OATP uptake transporters
Clarithromycin		Transporter mediated	Inhibition of P-gp efflux pump
Azithromycin		Transporter mediated	

**Table 2:** Potential enzyme-transporter-mediated drug-drug interactions of remdesivir (perpetrator) with anti-TB drugs (victim).

COVID Drug (Perpetrator)	Anti-TB Drugs (Victim)	Possible Pathway	Possible Mechanism
Remdesivir	Rifampin	Transporter/enzyme interplay	Inhibition of CYP3A4 enzyme and OATP uptake transporters
	PAS	Transporter mediated	Inhibition of OATP1B1 uptake transporter
	Clofazimine	Enzyme mediated	Inhibition of CYP1A2 enzyme
	Clarithromycin	Enzyme mediated	Inhibition of CYP3A4 enzyme
	Roxithromycin	Enzyme mediated	Inhibition of CYP3A4 enzyme
	Azithromycin	Enzyme mediated	Inhibition of OATP uptake transporters
	Bedaquiline	Enzyme mediated	Inhibition of CYP2C8 and CYP3A4 enzyme

**Table 3:** Potential enzyme-transporter-mediated drug-drug interactions of paxlovid (perpetrator) with anti-TB drugs (victim).

COVID Drug (Perpetrator)	Anti-TB Drugs (Victim)	Possible Pathway	Possible Mechanism
Paxlovid	Rifampin	Transporter/enzyme interplay	Inhibition of CYP3A4 enzyme and P-gp efflux transporters
	Rifabutin	Transporter/enzyme interplay	Inhibition of CYP3A4 enzyme and P-gp efflux transporters
	Rifapentine	Transporter/enzyme interplay	Inhibition of CYP3A4 enzyme and P-gp efflux transporters
	Clarithromycin	Enzyme mediated	Inhibition of CYP3A4 enzyme
	Roxithromycin	Enzyme mediated	Inhibition of CYP3A4 enzyme
	Clofazimine	Transporter mediated	Inhibition of P-gp uptake transporters
	Bedaquiline	Enzyme mediated	Inhibition of CYP3A4 enzyme

**Table 4:** Potential enzyme-transporter-mediated drug-drug interactions of paxlovid (victim) with anti-TB drugs (perpetrator).

COVID Drug (Victim)	Anti-TB Drugs (Perpetrator)	Possible Pathway	Possible Mechanism
Paxlovid	Rifampin	Transporter/enzyme interplay	Inhibition/induction of CYP3A4 enzyme and P-gp efflux transporters
	Prothionamide	Enzyme mediated	Inhibition of CYP3A4 enzyme
	Clofazimine	Transporter/enzyme interplay	Inhibition of CYP3A4, CYP2D6 enzyme and P-gp efflux transporters
	Clarithromycin	Enzyme mediated	Inhibition of CYP3A4 enzyme and P-gp efflux transporters
	Azithromycin	Transporter mediated	Inhibition of P-gp efflux transporters
	Linezolid	Enzyme mediated	Inhibition of CYP3A4 enzyme

**Table 5:** Drug-dependent model input parameters for the development of the PBPK-DDI model.

Parameters	Description	Data Source/Experimentation	Significance
Administration Route			
Intravenous, oral, dermal, etc., and associated parameters	Route and method of drug administration	Clinical trials	Determines the initial distribution and absorption characteristics of a drug
Formulation			
Dosage form	Physical form of the drug product	Pharmaceutical development studies	Influences drug release and absorption characteristics
Chemical Properties			
Molecular weight (MW)	Mass of a molecule	Analytical chemistry	Influences drug absorption, distribution, and elimination
Solubility/dissolution	Ability to dissolve in a solvent	Solubility assays, shake-flask method	Determines how well the drug dissolves in bodily fluids
Lipophilicity (LogP)	Measure of a compound’s affinity for lipid vs. water phases	LogP determination, HPLC, octanol-water partitioning	Affects membrane permeability and tissue distribution
pKa	Acid dissociation constant	pKa determination assays, potentiometric titration	Impacts ionization state, influencing absorption and distribution
Absorption Parameters			
Fraction unbound in plasma (f_u,p_)	Proportion of drug remains unbound in plasma	In vitro plasma binding studies or in silico modeling	Determines the PD and PK effects
Fraction unbound in gut (f_u,gut_)	Proportion of drug remains unbound in the gut	In vitro plasma binding studies or in silico modeling	Determines how much of the orally administered dose is available for absorption.
Absorption rate constants (K_a_)	Rate at which a drug enters systemic circulation	In vitro absorption studies (e.g., Caco-2 cells)	Determines the rate of drug absorption
Permeability coefficients (P_app_)	Measure of the drug’s ability to cross biological membranes	In vitro assays (e.g., Caco-2 cells, PAMPA)	Indicates the ability of the drug to cross biological membranes
Distribution Parameters			
Blood to Plasma ratio (B:P)	Ratio of concentrations of a compound in blood and plasma	Whole blood, equilibrium dialysis	Indicates how a drug distributes between plasma and blood
Tissue-to-plasma concentration ratios (K_p_)	Ratio of drug concentration in tissue to plasma	Imaging studies, literature data	Helps predict drug levels in different tissues
Metabolism Parameters			
Enzyme kinetic parameters (V_max_, K_m_)	Maximum rate of reaction and Michaelis constant	In vitro enzyme assays (e.g., microsomes, hepatocytes, recombinant enzyme)	Determines the rate of drug metabolism
Metabolic clearance rates	Rate at which a drug is metabolized	In vitro metabolism studies	Affects overall drug elimination from the body
Metabolism (Phase 1)			
Oxidation, reduction, hydrolysis (V_max_, K_m_)	Phase 1 metabolic reactions	In vitro enzyme assays, liver microsomes	Modifies drug structure to enhance excretion
Metabolism (Phase 2)			
Conjugation reactions (V_max_, K_m_)	Phase 2 metabolic reactions (e.g., glucuronidation, sulfation)	In vitro enzyme assays, liver microsomes	Increases drug solubility for easier excretion
Elimination Parameters			
Renal clearance (CL_r_)	Rate at which a drug is removed via the kidneys	Urine analysis	Determines the rate of drug removal via the kidneys
Hepatic clearance (CL_hep_)	Rate at which a drug is removed via the liver	Bile analysis	Determines the rate of drug removal via the liver
Clearance pathways	Routes of drug excretion (e.g., renal, biliary)	In vivo studies, literature data	Identifies routes of drug excretion
Transporters			
Membrane transporters (V_max_, K_m_)	Transport proteins that move drugs across cell membranes (e.g., P-gp, OATPs)	In vitro assays (e.g., cell lines expressing transporters)	Influence drug absorption, distribution, and elimination
DDI Model Parameters			
Inhibition constants (K_i_ or IC_50_)	Measure of a drug’s potency to inhibit enzymes or transporters	In vitro inhibition studies	Measures the potency of a drug to inhibit enzymes or transporters
Induction parameters (E_max_, EC_50_)	Maximum effect and concentration for half-maximal effect	In vitro induction studies, clinical studies	Measures the capacity of a drug to induce enzyme or transporter expression

## Data Availability

Data is contained within the article.

## References

[1] Gupta N., Ish P., Gupta A., Malhotra N., Caminero J.A., Singla R., Kumar R., Yadav S.R., Dev N., Agrawal S. (2020). A profile of a retrospective cohort of 22 patients with COVID-19 and active/treated tuberculosis. Eur. Respir. J..

[2] Tadolini M., Codecasa L.R., García-García J.M., Blanc F.X., Borisov S., Alffenaar J.W., Andréjak C., Bachez P., Bart P.A., Belilovski E. (2020). Active tuberculosis, sequelae and COVID-19 co-infection: First cohort of 49 cases. Eur. Respir. J..

[3] Beigel J.H., Tomashek K.M., Dodd L.E., Mehta A.K., Zingman B.S., Kalil A.C., Hohmann E., Chu H.Y., Luetkemeyer A., Kline S. (2020). Remdesivir for the Treatment of Covid-19—Final Report. N. Engl. J. Med..

[4] Aleissa M.M., Silverman E.A., Acosta L.M.P., Nutt C.T., Richterman A., Marty F.M. (2020). New Perspectives on Antimicrobial Agents: Remdesivir Treatment for COVID-19. Antimicrob. Agents Chemother..

[5] Fukunaga R., Glaziou P., Harris J.B., Date A., Floyd K., Kasaeva T. (2021). Epidemiology of Tuberculosis and Progress toward Meeting Global Targets—Worldwide, 2019. MMWR Morb. Mortal. Wkly. Rep..

[6] Zhang L., Lin D., Sun X., Curth U., Drosten C., Sauerhering L., Becker S., Rox K., Hilgenfeld R. (2020). Crystal structure of SARS-CoV-2 main protease provides a basis for design of improved α-ketoamide inhibitors. Science.

[7] Barnard D.L., Hubbard V.D., Burton J., Smee D.F., Morrey J.D., Otto M.J., Sidwell R.W. (2004). Inhibition of Severe Acute Respiratory Syndrome-Associated Coronavirus (SARSCoV) by Calpain Inhibitors and β-D-N4-Hydroxycytidine. Antivir. Chem. Chemother..

[8] Painter G.R., Natchus M.G., Cohen O., Holman W., Painter W.P. (2021). Developing a direct acting, orally available antiviral agent in a pandemic: The evolution of molnupiravir as a potential treatment for COVID-19. Curr. Opin. Virol..

[9] Riccardi N., Canetti D., Rodari P., Besozzi G., Saderi L., Dettori M., Codecasa L.R., Sotgiu G. (2021). Tuberculosis and pharmacological interactions: A narrative review. Curr. Res. Pharmacol. Drug Discov..

[10] Ogu C.C., Maxa J.L. (2000). Drug interactions due to cytochrome P450. Bayl. Univ. Med. Cent. Proc..

[11] Lynch T., Price A. (2007). The effect of cytochrome P450 metabolism on drug response, interactions, and adverse effects. Am. Fam. Physician.

[12] Bibi Z. (2008). Role of cytochrome P450 in drug interactions. Nutr. Metab..

[13] Rojas J.C., Aguilar B., Rodríguez-Maldonado E., Collados M.T. (2005). Pharmacogenetics of oral anticoagulants. Blood Coagul. Fibrinolysis.

[14] Sproule B.A., Otton S.V., Cheung S.W., Zhong X.H., Romach M.K., Sellers E.M. (1997). CYP2D6 inhibition in patients treated with sertraline. J. Clin. Psychopharmacol..

[15] Koepsell H. (2020). Organic Cation Transporters in Health and Disease. Pharmacol. Rev..

[16] Yonezawa A., Inui K.-I. (2011). Importance of the multidrug and toxin extrusion MATE/SLC47A family to pharmacokinetics, pharmacodynamics/toxicodynamics and pharmacogenomics. Br. J. Pharmacol..

[17] Pedersen J.M., Matsson P., Bergström C.A.S., Hoogstraate J., Norén A., LeCluyse E.L., Artursson P. (2013). Early Identification of Clinically Relevant Drug Interactions With the Human Bile Salt Export Pump (BSEP/ABCB11). Toxicol. Sci..

[18] Järvinen E., Deng F., Kiander W., Sinokki A., Kidron H., Sjöstedt N. (2021). The Role of Uptake and Efflux Transporters in the Disposition of Glucuronide and Sulfate Conjugates. Front. Pharmacol..

[19] Ho R.H., Tirona R.G., Leake B.F., Glaeser H., Lee W., Lemke C.J., Wang Y., Kim R.B. (2006). Drug and Bile Acid Transporters in Rosuvastatin Hepatic Uptake: Function, Expression, and Pharmacogenetics. Gastroenterology.

[20] Garrison D.A., Talebi Z., Eisenmann E.D., Sparreboom A., Baker S.D. (2020). Role of OATP1B1 and OATP1B3 in Drug-Drug Interactions Mediated by Tyrosine Kinase Inhibitors. Pharmaceutics.

[21] Telbisz Á., Ambrus C., Mózner O., Szabó E., Várady G., Bakos É., Sarkadi B., Özvegy-Laczka C. (2021). Interactions of Potential Anti-COVID-19 Compounds with Multispecific ABC and OATP Drug Transporters. Pharmaceutics.

[22] Humeniuk R., Mathias A., Kirby B.J., Lutz J.D., Cao H., Osinusi A., Babusis D., Porter D., Wei X., Ling J. (2021). Pharmacokinetic; Pharmacodynamic, and Drug-Interaction Profile of Remdesivir, a SARS-CoV-2 Replication Inhibitor. Clin. Pharmacokinet..

[23] Zanger U.M., Schwab M. (2013). Cytochrome P450 enzymes in drug metabolism: Regulation of gene expression, enzyme activities, and impact of genetic variation. Pharmacol. Ther..

[24] Lemaitre F., Solas C., Grégoire M., Lagarce L., Elens L., Polard E., Saint-Salvi B., Sommet A., Tod M., Guellec C.B.-L. (2020). Potential drug-drug interactions associated with drugs currently proposed for COVID-19 treatment in patients receiving other treatments. Fundam. Clin. Pharmacol..

[25] Atmar R.L., Finch N. (2022). New Perspectives on Antimicrobial Agents: Molnupiravir and Nirmatrelvir/Ritonavir for Treatment of COVID-19. Antimicrob. Agents Chemother..

[26] Mousquer G.T., Peres A., Fiegenbaum M. (2021). Pathology of TB/COVID-19 Co-Infection: The phantom menace. Tuberculosis.

[27] Foti R.S. (2024). Utility of PBPK Modeling in Predicting and Characterizing Clinical Drug Interactions. Drug Metab. Dispos..

[28] Owen D.R., Allerton C.M.N., Anderson A.S., Aschenbrenner L., Avery M., Berritt S., Boras B., Cardin R.D., Carlo A., Coffman K.J. (2021). An oral SARS-CoV-2 M(pro) inhibitor clinical candidate for the treatment of COVID-19. Science.

[29] Chatterjee B., Thakur S.S. (2022). Remdesivir and Its Combination With Repurposed Drugs as COVID-19 Therapeutics. Front. Immunol..

[30] Thomas L., Birangal S.R., Ray R., Miraj S.S., Munisamy M., Varma M., Sanju S.V. C., Banerjee M., Shenoy G.G., Rao M. (2021). Prediction of potential drug interactions between repurposed COVID-19 and antitubercular drugs: An integrational approach of drug information software and computational techniques data. Ther. Adv. Drug Saf..

[31] Rowland M., Peck C., Tucker G. (2011). Physiologically-based pharmacokinetics in drug development and regulatory science. Annu. Rev. Pharmacol. Toxicol..

[32] Jones H., Rowland-Yeo K. (2013). Basic Concepts in Physiologically Based Pharmacokinetic Modeling in Drug Discovery and Development. CPT Pharmacomet. Syst. Pharmacol..

[33] Luzon E., Blake K., Cole S., Nordmark A., Versantvoort C., Berglund E.G. (2017). Physiologically based pharmacokinetic modeling in regulatory decision-making at the European Medicines Agency. Clin. Pharmacol. Ther..

[34] Jamei M., Dickinson G.L., Rostami-Hodjegan A. (2009). A framework for assessing inter-individual variability in pharmacokinetics using virtual human populations and integrating general knowledge of physical chemistry, biology, anatomy, physiology and genetics: A tale of ‘bottom-up’ vs ‘top-down’ recognition of covariates. Drug Metab. Pharmacokinet..

[35] Grimstein M., Yang Y., Zhang X., Grillo J., Huang S.M., Zineh I., Wang Y. (2019). Physiologically Based Pharmacokinetic Modeling in Regulatory Science: An Update From the U.S. Food and Drug Administration’s Office of Clinical Pharmacology. J. Pharm. Sci..

[36] European Medicines Agency (2021). Regulatory Guidelines on the Reporting of Physiologically Based Pharmacokinetic (Pbpk) Modeling Analysis, Physiologically Based Pharmacokinetic (PBPK) Modeling and Simulations.

[37] Palleria C., Di Paolo A., Giofrè C., Caglioti C., Leuzzi G., Siniscalchi A., De Sarro G., Gallelli L. (2013). Pharmacokinetic drug-drug interaction and their implication in clinical management. J. Res. Med. Sci..

[38] Li C., Chen L., Li L., Chen W. (2023). Drug-drug interactions and dose management of BTK inhibitors when initiating nirmatrelvir/ritonavir (paxlovid) based on physiologically-based pharmacokinetic models. Eur. J. Pharm. Sci..

[39] Sagawa K., Lin J., Jaini R., Di L. (2023). Physiologically-Based Pharmacokinetic Modeling of PAXLOVID™ with First-Order Absorption Kinetics. Pharm. Res..

[40] Wang Z., Chan E.C.Y. (2022). Physiologically-Based Pharmacokinetic Modeling-Guided Dose Management of Oral Anticoagulants when Initiating Nirmatrelvir/Ritonavir (Paxlovid) for COVID-19 Treatment. Clin. Pharmacol. Ther..

[41] Deb S., Reeves A.A. (2021). Simulation of Remdesivir Pharmacokinetics and Its Drug Interactions. J. Pharm. Pharm. Sci..

[42] Jamei M. (2020). Where Do PBPK Models Stand in Pharmacometrics and Systems Pharmacology?. CPT Pharmacomet. Syst. Pharmacol..

[43] Min J.S., Bae S.K. (2017). Prediction of drug-drug interaction potential using physiologically based pharmacokinetic modeling. Arch. Pharm. Res..

[44] Lin W., Chen Y., Unadkat J.D., Zhang X., Wu D., Heimbach T. (2022). Applications, Challenges, and Outlook for PBPK Modeling and Simulation: A Regulatory, Industrial and Academic Perspective. Pharm. Res..

[45] Mitra A., Parrott N., Miller N., Lloyd R., Tistaert C., Heimbach T., Ji Y., Kesisoglou F. (2020). Prediction of pH-Dependent Drug-Drug Interactions for Basic Drugs Using Physiologically Based Biopharmaceutics Modeling: Industry Case Studies. J. Pharm. Sci..

[46] Salem F., Nimavardi A., Mudunuru J., Tompson D., Bloomer J., Turner D.B., Taskar K.S. (2023). Physiologically based pharmacokinetic modeling for development and applications of a virtual celiac disease population using felodipine as a model drug. CPT Pharmacomet. Syst. Pharmacol..

[47] Gill J., Moullet M., Martinsson A., Miljković F., Williamson B., Arends R.H., Reddy V.P. (2023). Evaluating the performance of machine-learning regression models for pharmacokinetic drug-drug interactions. CPT Pharmacomet. Syst. Pharmacol..

[48] Taskar K.S., Reddy V.P., Burt H., Posada M.M., Varma M., Zheng M., Ullah M., Riedmaier A.E., Umehara K.I., Snoeys J. (2020). Physiologically-Based Pharmacokinetic Models for Evaluating Membrane Transporter Mediated Drug-Drug Interactions: Current Capabilities, Case Studies, Future Opportunities, and Recommendations. Clin. Pharmacol. Ther..

